# *L*-DOPA decarboxylase mRNA expression is associated with tumor stage and size in head and neck squamous cell carcinoma: a retrospective cohort study

**DOI:** 10.1186/1471-2407-12-484

**Published:** 2012-10-20

**Authors:** Panagiota-Aikaterini Geomela, Christos K Kontos, Ioannis Yiotakis, Emmanuel G Fragoulis, Andreas Scorilas

**Affiliations:** 1 Department of Biochemistry and Molecular Biology, University of Athens, Panepistimiopolis, Athens 15701, Greece; 2 First Ear, Nose & Throat Clinics, Athens General Hospital ‘Hippokration, University of Athens, 114 Vasilissis Sofias Ave, Athens 11527, Greece

**Keywords:** Oral cancer, Larynx, Tongue, Tumor biomarkers, Quantitative real-time PCR

## Abstract

**Background:**

Head and neck squamous cell carcinoma (HNSCC) represents one of the most commonly diagnosed malignancies worldwide. The *DDC* gene encodes *L*-DOPA decarboxylase, an enzyme catalyzing the decarboxylation of *L*-DOPA to dopamine. We have recently shown that *DDC* mRNA is a significant predictor of patients’ prognosis in colorectal adenocarcinoma and prostate cancer. The aim of the current study was to analyze the *DDC* mRNA expression in HNSCC patients.

**Methods:**

53 malignant tumors were resected from the larynx, pharynx, tongue, buccal mucosa, parotid glands, and nasal cavity, as well as from 34 adjacent non-cancerous tissues of HNSCC patients, and were homogenized. Total RNA was isolated and converted into first-strand cDNA. An ultrasensitive real-time PCR method based on the SYBR Green chemistry was used for *DDC* mRNA quantification in head and neck tissue specimens. Relative quantification was performed using the comparative Ct (2^-ddCt^) method.

**Results:**

*DDC* mRNA levels were lower in squamous cell carcinomas (SCCs) of the larynx and tongue than in adjacent non-cancerous tissue specimens. Furthermore, low *DDC* mRNA expression was noticed in laryngeal and tongue tumors of advanced TNM stage or bigger size, compared to early-stage or smaller tumors, respectively. No statistically significant differences were observed between SCCs resected from pharynx, buccal mucosa, or nasal cavity, and their normal counterparts.

**Conclusion:**

This is the first study examining the *DDC* mRNA expression in HNSCC. According to our results, *DDC* mRNA expression may constitute a potential prognostic biomarker in tongue and/or larynx SCCs, which principally represent the overwhelming majority of HNSCC cases.

## Background

The most usual malignancies developed in the head and neck area fall into the category of squamous cell carcinomas (SCCs) [1]. Head and neck squamous cell carcinoma (HNSCC) holds a remarkable position among the causes leading to death in the developed world [2]. In particular, this disease is endemic to Southern China and Southeast Asian countries, whereas its incidence is significantly low in Europe and North America, though there is evidence of an increasing occurrence even in these areas [3]. An intermediate incidence has also been reported in Alaskan Eskimos and the Mediterranean Basin (North Africa, South Italy, Greece, and Turkey) [4]. This diversification in the HNSCC incidence among populations of distinct geographic areas implies a strong association with genetic and environmental factors [5].

HNSCC includes a great variety of tumors originating from epithelial cells lining different sites of the head and neck region. Squamous cell carcinomas arise in nasal cavity and paranasal sinuses, nasopharynx, hypolarynx, larynx and trachea, oral cavity, oropharynx, salivary glands, and ear, while miscellaneous tumors such as neurogenic neoplasms and paragangliomas may also appear [6]. The molecular basis of HNSCC is very complex and extremely heterogeneous, exhibiting remarkable differences even among people with tumors of the same type and stage. Research conducted during the last decade has shed light to the molecular mechanisms underlying HNSCC; yet, many essential pieces are still missing from the puzzle [2]. The discovery and prospective evaluation of novel molecular biomarkers constitute a big challenge for the scientific community [7].

The human gene encoding *L*-DOPA decarboxylase (DDC) maps to chromosome 7p12.1–12.3, close to the epidermal growth factor receptor (*EGFR*) gene, and is composed of 15 exons spanning a genomic region of more than 85 kb [8]. Multiple alternatively spliced variants of the *DDC* gene have been described, with most of them encoding distinct protein isoforms [9-12]. DDC is a pyridoxal-phosphate (PLP)-dependent enzyme catalyzing the decarboxylation of 3,4-dihydroxy-*L*-phenylalanine (*L*-DOPA) to dopamine and 5-hydroxy-*L*-tryptophan (5-HTP) to serotonin [13]. DDC is localized both in the cytosol and cell membrane [14]. Enzymatically active DDC has already been detected in brain, liver, kidney, adrenal glands, pancreas, cervix [15], placenta [16], as well as in peripheral leukocytes and T-cells [17]. The presence of DDC activity in leukocytes and in the histiocytic lymphoma cell line U-937 implies a cross-talk between the nervous and the immune system, and raises new questions about the regulatory role of DDC in immune responses [18,19]. DDC is also a coregulator of the androgen receptor (AR) in prostate cells. In more detail, DDC exerts its function in the cytoplasm by enhancing the AR activity, and hence modulates the expression of AR-regulated genes [20,21]. Recently, the identification and purification of two novel inhibitors of the enzymatic DDC activity have been reported. One inhibitor has been isolated from the human serum and the other from the membrane fraction of human placental tissue. [22,23]. Although both inhibitors are believed to have an endogenous function, their physiological role remains to be elucidated.

Notably, DDC is regarded as a general biomarker for neuroendocrine malignancies [24-26]. For instance, elevated *DDC* mRNA expression has been detected in small-cell lung carcinoma [27,28] and neuroblastoma [29]. It has been postulated that *DDC* mRNA expression constitutes a biomarker for the detection of minimal residual disease (MRD) in neuroblastoma patients, as well as a useful biomarker for the discrimination of neuroblastoma from other small round-cell malignancies of childhood [29,30]. Data from our lab support also the notion that *DDC* mRNA expression could be used as a new tissue biomarker in prostate cancer [31], as it can reliably predict biochemical recurrence and shorter disease-free survival (DFS) interval in prostate cancer patients who have previously been subjected to radical prostatectomy [32]. In addition, Sakakura *et al.* showed that *DDC* is overexpressed in peritoneal dissemination of gastric carcinoma, and suggested that *DDC* mRNA expression is potentially a novel biomarker for the detection of peritoneal metastases [33].

Interestingly, DDC is implicated in the pathobiology of prostate cancer, since it promotes abnormal prostate cell proliferation and neuroendocrine differentiation in an AR-dependent manner [34]. Moreover, DDC seems to play a major role in cancer pathobiology and progression, since it catalyzes the synthesis of biogenic amines participating in angiogenesis, cell proliferation, and differentiation [35,36]. Dopamine as well as other catecholamines inhibit erythrocyte apoptosis by preventing scramblase activation and subsequent phosphatidylserine exposure on the cell membrane [37], which in turn triggers the clearance of apoptotic cells by macrophages.

The aforementioned data prompted us to analyze *DDC* mRNA expression in HNSCC and adjacent non-cancerous tissue specimens resected from patients having malignant tumors in larynx, pharynx, tongue, buccal mucosa, parotid glands, or nasal cavity, with the use of an hypersensitive quantitative real-time PCR (qRT-PCR) method based on the SYBR Green chemistry, and to evaluate its clinical significance and application as a novel tissue biomarker for HNSCC.

## Methods

### Patients’ tissue specimens

A total of 53 malignant tumors and 34 adjacent non-cancerous tissue specimens from patients having undergone surgical treatment for primary HNSCC at Athens General Hospital “Hippokration” (Athens, Greece) between 2005 and 2007, were included in the current study. Tissue specimens were resected from larynx (20 cases), pharynx (5 cases), tongue (14 cases), buccal mucosa (5 cases), parotid glands (5 cases), and nasal cavity (4 cases). The age of the patients included in this study varied from 34.0 to 90.0 years, with a mean ± SE of 63.1 ± 1.6. All specimens incorporated in the study were selected after having taken into account the availability of sufficient tissue mass for RNA extraction and assay, while they had been frozen in liquid nitrogen immediately after their resection.

The present study was conducted in accordance with the ethical standards of the World Medical Association Declaration of Helsinki (version: 2008), and was approved by the institutional review board of Athens General Hospital “Hippokration” (Athens, Greece). Moreover, informed consent was obtained from HNSCC patients participating in this study.

### RNA extraction and reverse transcription

Tissue specimens were pulverized and then dissolved in TRI Reagent (Ambion Europe Ltd., Huntingdon, UK). Following the manufacturer’s instructions, total RNA was extracted and diluted in RNA Storage Solution (Ambion Europe Ltd.), and stored at -80^o^C until use. First-strand cDNA was then synthesized using the M-MuLV Reverse Transcriptase, RNase H^–^ (Finnzymes Oy, Vantaa, Finland), RNaseOUT RNase inhibitor (Invitrogen, Carlsbad, CA, USA), and oligo(dT)_12-18_ as primer, according to the manufacturer’s instructions.

### Quantitative real-time PCR (qRT-PCR)

Taking into account the sequences of the *DDC* and *GAPDH* cDNA (GenBank Accession Numbers: NM_000790 and NM_002046, respectively), we designed two pairs of gene-specific primers. The *DDC* primers anneal to all *DDC* transcripts except for the *alt-DDC* variant, which possesses an alternative C-terminus [10], and give birth to a single amplicon. The sequences of the *DDC* and *GAPDH* real-time PCR primers, the lengths of the PCR amplicons, and their melting temperatures (T_m_) are shown in Table 1. Quantitative real-time PCR (qRT-PCR) was accomplished on a 7500 Real Time PCR System (Applied Biosystems, Foster City, CA, USA) using the SYBR Green chemistry, and dissociation curves of the PCR products were next produced, as previously described [38], allowing for the discrimination of the main PCR products from primer-dimers or other non-specific products. Each real-time PCR reaction was conducted in duplicate, in order to evaluate data reproducibility.

**Table 1:** **Primers used for real-time PCR amplification of *DDC *and *GAPDH***

**Gene**	**Primer sequence**	**Length of the PCR product (bp)**	**T**_**m**_**of the PCR product (**^**o**^**C)**
***DDC***	5′-GAACAGACTTAACGGGAGCCTTT-3^′^	90	79.0
	5′-AATGCCGGTAGTCAGTGATAAGC-3^′^		
***GAPDH***	5′-ATGGGGAAGGTGAAGGTCG-3^′^	107	79.4
	5′-GGGTCATTGATGGCAACAATATC-3^′^		

Calculations took place with the use of the comparative Ct (2^-ddCt^) method [39]. In our study, *GAPDH* played the role of an internal control gene for the normalization of the PCRs for the amount of RNA added to the first-strand cDNA synthesis reactions, while the colorectal adenocarcinoma epithelial cell line Caco-2 served as a calibrator. ddCt stands for the difference between the mean dCt value of a HNSCC sample and the mean dCt of the calibrator, both calculated after the same PCR run, while dCt represents the difference between the threshold cycle (Ct) of the target gene (*DDC*) and the Ct of the reference gene (*GAPDH*) of the same sample. Normalized results were expressed as arbitrary units (a.u.), standing for the ratio of *DDC* mRNA copies to 1000 *GAPDH* mRNA copies calculated for each specimen, in relation to the same ratio calculated for Caco-2 cells.

### Statistical analysis

Advanced biostatistical analysis was carried out only with regard to the laryngeal squamous cell carcinoma (LSCC) and tongue squamous cell carcinoma (TSCC) specimens, since the small number of cases in the rest HNSCC patients’ groups did not allow an extensive analysis to be carried out. LSCC and TSCC patients were classified in subgroups according to the TNM stage, the histological grade, and the size of their tumors; *DDC* mRNA levels in these subgroups were compared using the non-parametric Mann-Whitney *U* or Kruskal-Wallis test, where appropriate.

## Results

### Validation of the comparative Ct (2^-ddCt^) method for *DDC* mRNA quantification

**Figure 1:**
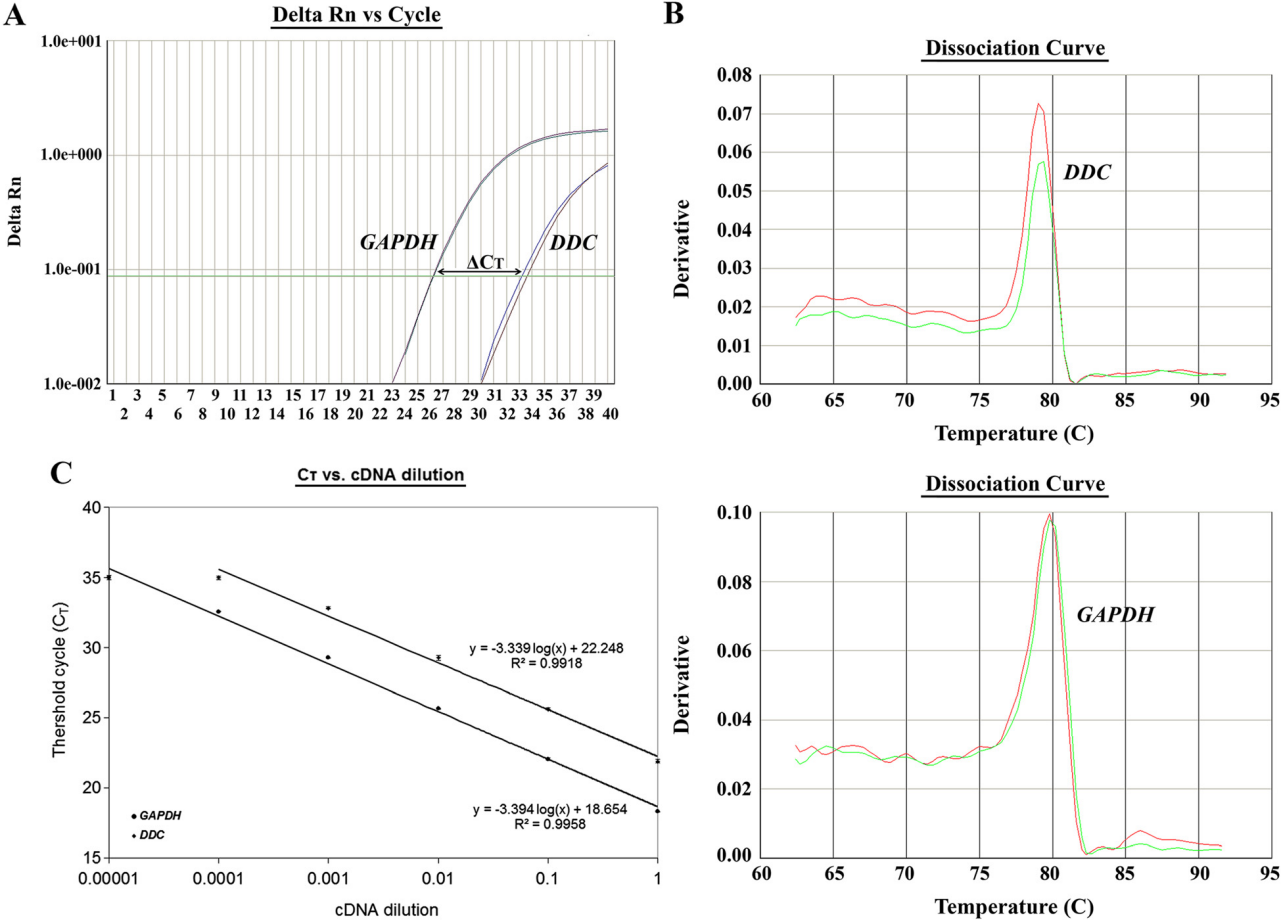
**Relative quantification of *DDC *mRNA expression in a laryngeal squamous cell carcinoma specimen, using qRT-PCR.** (**A**) Amplification plots of *DDC* and *GAPDH* cDNAs, (**B**) dissociation curves of the respective amplicons, showing dRn plotted against cycle number, and (**C**) validation of the comparative Ct (2^-ddCt^) method, to evaluate the efficiency of *DDC* and *GAPDH* amplification.

The validation of the comparative Ct (2^-ddCt^) method, in order to be feasible, requires two presuppositions. More specifically, the PCR amplification efficiencies of the target and the reference genes should approximate to 100% and be quite equal to each other [39]. In the present study, both prerequisites were tested in a validation experiment, in which the Ct values of *DDC* and *GAPDH* cDNA amplification were determined in a dilution series of Caco-2 cDNA over a 10^4^-fold range and then plotted against the log cDNA dilution. The Ct values corresponded to the number of cycles at which the fluorescence emission monitored in real time reached a threshold of 10 times the standard deviation of the mean baseline emission from cycles 3 to 15 (Figure 1A). It should be added that all PCR products were gene-specific, as illustrated by the dissociation curves (Figure 1B). The slopes of *DDC* and *GAPDH* amplification plots, as illustrated in Figure 1C, are similar (-3.339 and -3.394, respectively). Applying the formula *E(%)* = [-1 + 10^(-1/α)^^.^100, where *E(%)* is the real-time PCR efficiency for amplification of each gene and α is the slope of the corresponding amplification plot, the values of the efficiencies of *DDC* and *GAPDH* PCR amplification were 99.3% and 97.1%, respectively. Therefore, both prerequisites for the application of the 2^-ddCt^ method were satisfied.

### *DDC* mRNA expression analysis in cancerous and non-cancerous specimens of the head and neck

*DDC* mRNA expression was lower in LSCC specimens than in non-malignant counterparts, varying between 0.05 and 164.91 a.u. with a mean±S.E. of 17.50 ± 11.52 a.u. in the former, while ranging from 0.05 to 359.72 a.u. with a mean±S.E. of 44.02 ± 25.71 a.u. in the latter (Table 2). Similarly, *DDC* mRNA expression levels presented a slight decrease in TSCC specimens, in comparison with their non-malignant counterparts. Therefore, *DDC* mRNA levels in laryngeal tumors fluctuated between 0.05 and 93.9 a.u. with a mean±SE of 7.43 ± 7.21, whereas in non-malignant laryngeal tissue specimens they ranged from 0.05 to 128.80 a.u. with a mean±SE of 24.55 ± 16.99 (Table 2).

**Table 2:** ***DDC *mRNA expression**^**a**^**analysis in patients with laryngeal or tongue tumors and their non-cancerous counterparts**

**Variable**	**Mean ± SE**^**b**^	**Range**	**Percentiles**				
			**10**	**25**	**50**	**75**	**90**
					**Median**		
*DDC* in laryngeal tumors (N = 19)	17.50 ± 11.52	0.05 – 164.91	0.05	0.05	0.05	0.52	153.89
*DDC* in non-cancerous laryngeal tissues (N = 14)	44.02 ± 25.71	0.05 – 359.72	0.05	0.05	3.71	61.61	228.09
*DDC* in tongue tumors (N = 13)	7.43 ± 7.21	0.05 – 93.9	0.05	0.05	0.05	0.63	56.77
*DDC* in non-cancerous tongue tissues (N = 8)	24.55 ± 16.99	0.05 – 128.80	0.05	0.05	0.05	49.92	128.80

^a^ a.u.: arbitrary units.

^b^ Standard error of the mean.

Table 3 shows features of the distribution of *DDC* mRNA expression in tumors resected from pharynx, buccal mucosa, parotid glands, and nasal cavity. Because of the small number of cases of each group, only descriptive statistical analysis was performed for these results. Thus, *DDC* mRNA levels seem to be lower in tumors of the nasal cavity than in non-cancerous counterparts. In contrast, higher levels of *DDC* transcripts were detected in malignant neoplasms of pharynx and parotid glands, compared to their non-malignant counterparts.

**Table 3:** **Distribution of *DDC*mRNA expression**^**a**^**in head and neck squamous cell carcinomas and in non-cancerous counterparts**

**Tissue**	**Cancerous samples**			**Non-cancerous samples**		
	**Mean ± SE**^**b**^	**Median**	**Range**	**Mean ± SE**^**b**^	**Median**	**Range**
Pharynx	5.51 ± 4.66	0.20	0.05 – 23.98	4.21 ± 3.5	1.07	0.05 – 14.65
Buccal mucosa	2.71 ± 2.66	0.05	0.05 – 13.33	-	-	-
Parotid glands	2.23 ± 1.88	0.10	0.05 – 9.69	0.31 ± 0.26	0.05	0.05 – 1.07
Nasal cavity	0.39 ± 0.24	0.23	0.05 – 1.05	8.75 ± 8.69	0.05	0.05 – 26.13

^a^ a.u.: arbitrary units.

^b^ Standard error of the mean.

### *DDC* mRNA expression analysis in subgroups of LSCC and TSCC patients

LSCC and TSCC groups of patients were further classified in subgroups, according to classical clinicopathological parameters, such as tumor size, TNM stage, and histological grade. *DDC* mRNA expression analysis was then conducted for each subgroup, and *DDC* transcription levels were compared among distinct patients’ subgroups using the non-parametric Mann-Whitney *U* or Kruskal-Wallis test, as described in the “Methods” section.

Concerning LSCC patients, those bearing small tumors exhibited remarkably increased levels of *DDC* mRNA, in contrast to patients with bigger tumors (p = 0.033). In particular, in patients with tumor size ≤2 cm, *DDC* expression showed a mean±S.E. of 40.95 ± 25.88 a.u., whereas in patients with tumor size >2 cm the respective mean±S.E. was 0.42 ± 0.37 (Table 4). LSCC patients being at an early disease stage (TNM stage I) displayed, also, very elevated *DDC* expression, in comparison with patients diagnosed at an intermediate (TNM stage II) or an advanced stage (TNM stage III) (p = 0.027). On the other hand, no statistically significant difference was noticed between subgroups of TSCC patients classified according to the histological grade of the tumor.

**Table 4:** ***DDC *mRNA expression**^**a**^**analysis in subgroups of LSCC patients, classified according to classical clinicopathological parameters**

**Variable**	**No. of patients**	**Mean ± SE**^**b**^	**Median**	**p value**
Tumor size				
≤ 2 cm	8	40.95 ± 25.88	0.41	0.033^c^
> 2 cm	11	0.42 ± 0.37	0.05	
TNM stage				
I	5	63.94 ± 39.01	0.52	0.027^d^
II	3	2.65 ± 2.61	0.05	
III	11	0.42 ± 0.37	0.05	
Histological grade				
I	7	0.12 ± 0.07	0.05	0.46^d^
II	6	28.22 ± 27.35	0.05	
III	6	27.03 ± 25.40	0.17	

^a^ a.u.: arbitrary units.

^b^ Standard error of the mean.

^c^ Calculated using the Mann-Whitney *U* test.

^d^ Calculated using the Kruskal-Wallis test.

Similarly, *DDC* mRNA expression was found to be 2.7-fold higher in tongue tumors of ≤2 cm than in bigger tumors (p = 0.028). A dramatic decrease in *DDC* gene transcription was also detected in intermediate- and late-stage TSCC patients (p = 0.025). In more detail, in patients with TNM stage I tongue SCC, *DDC* expression had a mean±S.E. of 31.73 ± 3.11 a.u., while in patients with tongue SCC of TNM stage II and III the mean±S.E. was 0.18 ± 0.13 and 0.05 ± 0.003, respectively (Table 5). No statistically significant differences were observed between subgroups of TSCC patients classified according to the tumor histological grade.

**Table 5:** ***DDC *mRNA expression**^**a**^**analysis in subgroups of TSCC patients, classified according to classical clinicopathological parameters**

**Variable**	**No. of patients**	**Mean ± SE**^**b**^	**Median**	**p value**
Tumor size				
≤ 2 cm	7	13.76 ± 3.36	0.26	0.028^c^
> 2 cm	6	5.08 ± 0.02	0.05	
TNM stage				
I	3	31.73 ± 3.11	1.00	0.025^d^
II	7	0.18 ± 0.13	0.05	
III	3	0.05 ± 0.003	0.05	
Histological grade				
I	5	0.24 ± 0.19	0.05	0.097^d^
II	4	0.28 ± 0.23	0.05	
III	3	31.34 ± 31.29	0.05	

^a^ a.u.: arbitrary units.

^b^ Standard error of the mean.

^c^ Calculated using the Mann-Whitney *U* test.

^d^ Calculated using the Kruskal-Wallis test.

## Discussion

HNSCC today represents the sixth most common malignancy affecting people in the developed countries, while the largest proportion of the cases arise in the areas of larynx and the oral cavity [1]. The leading cause of the HNSCC is predominately tobacco and alcohol abuse. Other factors which enhance the possibility of developing such tumors are the family history of the disease, which is indicative of genetic predisposition, virus infections such as HPV (human papillomavirus), EBV (Epstein-Barr virus), HSV (Herpes simplex virus) and HIV (human immunodeficiency virus), as well as an unhealthy diet and exposure to carcinogens on a permanent base, e.g. an unsanitary professional environment [3].

Undoubtedly, 5-year survival rates of advanced laryngeal and pharyngeal carcinoma patients have improved a lot during the last decade, mostly thanks to the novel therapeutic approaches that have emerged; still, the overall survival (OS) of HNSCC patients remains among the lowest. Usually, treatment strategies fail or procure rather modest improvement and consequently, locoregional recurrence, distant metastases, and second primary tumors are frequent. In particular, positive nodal status is considered as the most adverse independent prognosticator in HNSCC [7]. Despite the recent advances, the morbidity and the mortality attributed to this disease remain among the highest [2]. The poor prognosis of HNSCC patients is mostly due to lack of established biomarkers for early detection and treatment monitoring [40]. Taking all this information into consideration, the discovery of novel, reliable molecular biomarkers for HNSCC may contribute to the prolongation of patients’ survival through early diagnosis, reliable prognosis and/or effective treatment response monitoring, while it may also illuminate the molecular background of this cancer type [41].

To date, several molecules have been evaluated and proposed as potential biomarkers in HNSCC, including *EGFR*, cyclin D1 (CCND1), Ki67 antigen, FAS, FASL, *BCL2*, *BCL2L12*, TP53, P27, vascular endothelial growth factor (VEGF), matrix metallopeptidases (MMPs), and kallikrein-related peptidases (*KLK*s) [7,42-46]. Our study introduces the mRNA expression of *DDC* as a new promising tumor biomarker for HNSCC. *DDC* mRNA expression has already been demonstrated to increase in many tissues; modulations in its expression levels have been reported in various malignancies, such as neuroblastoma [30], small cell lung carcinoma [16], and prostate cancer [20,31]. Most interestingly, strong DDC mRNA expression has been detected in tumors of the gastrointestinal tract [33,38].

Extremely high DDC activity in neoplastic cells is a hallmark of several peripheral cancers. This is mostly apparent in lung tumors of small-cell origin, although nonsense alternatively spliced variants are also present [35]. Furthermore, a remarkable increase in DDC activity has been detected in primary intestinal cancer as well as in its related metastases in the spleen and liver, in comparison with normal tissue [25]. The significance of elevated DDC activity and increased monoamine synthesis by the neoplastic cells remains to be elucidated [35]; still, it is closely related to the involvement of DDC in cancer.

In this study, we undertook the quantitative expression analysis of *DDC* mRNA in SCCs of the larynx, pharynx, tongue, buccal mucosa, parotid glands, and nasal cavity, as well as in adjacent non-cancerous counterparts. Our results revealed that *DDC* mRNA levels are 2.5- and 3.3-fold lower in laryngeal and tongue tumors, respectively, than in non-cancerous adjacent tissues. Moreover, *DDC* mRNA expression differs significantly in LSCC and TSCC patients, when classified according to the TNM stage or size of malignant tumors. Concretely, LSCC patients bearing tumors of early TNM stage and/or smaller than 2 cm displayed higher *DDC* mRNA expression than patients with more advanced tumors. The decreased *DDC* mRNA expression that seems to be associated with progression of HNSCC could imply a protective role of DDC against HNSCC, perhaps owing to its indirect association with apoptosis [37]. In spite of the fact that clinical comorbidities have been shown to significantly affect survival over TNM prognosticators [47], the TNM classification consistently correlates with DFS and OS [48]. Given that the TNM staging of LSCC remains the most significant predictor of survival and that *DDC* mRNA levels are high in early-stage patients, *DDC* could constitute a favorable prognostic indicator in LSCC. Moreover, *DDC* has a similar expression pattern in colorectal adenocarcinoma, possessing favorable prognostic value. Elevated *DDC* mRNA expression levels were found in well-differentiated and early-stage colorectal adenocarcinomas, and were shown to predict better patient outcome, in terms of DFS and OS [38]. The prognostic potential of *DDC* mRNA status in prostate cancer has also recently been uncovered. In more detail, *DDC* mRNA is likely to constitute an unfavorable prognosticator in this malignancy, predicting biochemical recurrence and poor DFS in prostate cancer patients treated by radical prostatectomy [32]. Additionally, *DDC* mRNA levels were shown to be significantly higher in prostate cancer than in benign prostate hyperplasia patients. Therefore, *DDC* mRNA status seems to possess significant discriminatory value in this malignancy [31].

On the other hand, increased *DDC* expression is associated with peritoneal metastasis, since its mRNA is upregulated in gastric cancer peritoneal dissemination [33]. This discrepancy could be attributed to the utilization of distinct gene promoters [49], which regulate the expression of this gene in a tissue-specific manner [50]. In fact, several molecules have been shown to be overexpressed in various malignancies, while being downregulated in other types of cancer. For instance, *BCL2L12*, a member of the BCL2 family of apoptosis-related genes, is overexpressed in undifferentiated nasopharyngeal carcinoma [43] and in poorly differentiated TSCC, whereas its mRNA levels are lower in LSCC of advanced TNM stage, compared to early-stage laryngeal tumors [45].

In this framework, the potential correlation between *DDC* mRNA expression and DFS and/or OS of LSCC patients deserves further investigation. Hence, our future goals include *DDC* mRNA expression analysis in larger cohorts of LSCC and TSCC samples in order to validate the current results, since the number of SCC specimens used in this study constitutes a limiting factor, owing to the fact that HNSCC is not a very common cancer type in Greece [51]. Furthermore, the statistically significant correlation of *DDC* expression with the tumor size and the TNM stage of the disease, as confirmed in LSCC and TSCC patients, might contribute to more accurate and effective staging of laryngeal and tongue tumors, respectively. The implementation of molecular criteria in disease staging may aid to distinguish between neoplasms which, though different at the molecular level, are grouped together, based on the current morphological and clinicopathological parameters. To sum up, this study proposes *DDC* mRNA expression as a useful biomarker for better classification of the laryngeal and tongue tumors. Although mRNA levels are heavily dependent on ribonuclease activity, *DDC* mRNA expression, if combined with other putative biomarkers, could add to their prognostic value or be part of a multiparametric model significantly assisting with HNSCC classification, prognosis, and response to chemotherapy.

## Conclusion

To the best of our knowledge, this is the first time that this gene is studied in HNSCC. Our results imply the clinical usefulness of *DDC* mRNA expression as a potential prognostic biomarker in TSCC and/or LSCC, which principally compose the vast majority of HNSCC cases. Additional studies could shed light to the molecular mechanisms underlying the role of DDC in HNSCC progression, and thoroughly examine the prognostic value of *DDC* mRNA expression in LSCC, TSCC, and other types of HNSCC. With regard to our future goals, we are planning to compare the *DDC* mRNA expression profile in a larger cohort of LSCC and TSCC patients, and in healthy controls. The expression analysis will also discriminate between distinct variants of the *DDC* gene. Furthermore, we intend to assess the prognostic value of this potential biomarker in HNSCC and to examine the effect of widely used chemotherapy on *DDC* expression.

## Abbreviations

SCC: Squamous cell carcinoma; HNSCC: Head and neck squamous cell carcinoma; DDC: *L*-DOPA decarboxylase; EGFR: Epidermal growth factor receptor; PLP: Pyridoxal-phosphate; *L*-DOPA: 3:4-dihydroxy-*L*-phenylalanine; 5-HTP: 5-hydroxy-*L*-tryptophan; AR: Androgen receptor; MRD: Minimal residual disease; qRT-PCR: Quantitative real-time PCR; cDNA: DNA complementary to RNA; RNase: Ribonuclease; Oligo(dT): Oligodeoxythymidine; GAPDH: Glyceraldehyde-3-phosphate dehydrogenase; a.u: Arbitrary units; LSCC: Laryngeal squamous cell carcinoma; TSCC: Tongue squamous cell carcinoma; HPV: Human papillomavirus; EBV: Epstein-Barr virus; HSV: Herpes simplex virus; HIV: Human immunodeficiency virus; OS: Overall survival; DFS: Disease-free survival.

## Competing interests

The authors declare that they have no competing interests.

## Authors’ contributions

PAG carried out the experimental work, collected and analyzed data, and drafted the manuscript. CKK interpreted the results and drafted the manuscript. IY designed the study, collected patients’ material and follow-up data. EGF revised critically the manuscript. AS conceived of the study, coordinated the study, and performed the statistical analysis. All authors read and approved the final manuscript.

## Pre-publication history

The pre-publication history for this paper can be accessed here:

http://www.biomedcentral.com/1471-2407/12/484/prepub
